# Draft Genome Sequence of *Rhodococcus ruber* Strain BKS 20-38

**DOI:** 10.1128/genomeA.00139-13

**Published:** 2013-04-04

**Authors:** Monu Bala, Shailesh Kumar, Gajendra Pal Singh Raghava, Shanmugam Mayilraj

**Affiliations:** Microbial Type Culture Collection and Gene Bank (MTCC)a and; Bioinformatics Centre,b CSIR-Institute of Microbial Technology, Chandigarh, India

## Abstract

We report the 6.1-Mb genome sequence of *Rhodococcus ruber* strain BKS 20-38, isolated from the palm tree rhizosphere soil of Bhitarkanika National Park, Odhisha, India. The draft genome sequence of strain BKS 20-38 consists of 6,126,900 bp, with a G+C content of 69.72%, 5,716 protein-coding genes, and 49 RNAs.

## GENOME ANNOUNCEMENT

The genus name *Rhodococcus* was first proposed by Zopf (1891) and emended by Tsukamura (1974) (1) and Goodfellow and Alderson (1977) (2). *Rhodococcus ruber* was first proposed by Kruse (1896) and later on emended by Goodfellow and Alderson (1977) (2). We have isolated the *Rhodococcus ruber* strain BKS 20-38 from palm tree rhizosphere soil from Bhitarkanika National Park, Odhisha, India. This strain shows cholesterol oxidase activity, which degrades cholesterol into 4-cholesten-3-one. The genome of *Rhodococcus ruber* strain BKS 20-38 was sequenced using the Illumina-HiSeq 1000 paired-end technology, which produced a total of 65,135,348 paired-end reads (insert size of 350 bp) of 101 bp. We used NGS QC Toolkit v2.3 (3) to filter the data for high-quality, vector/adaptor-free reads for genome assembly (cutoff read length for HQ, 70%; cutoff quality score, 20). A total of 60,504,470 high-quality, vector-filtered reads (~1,018.5-fold coverage) were used for assembly with SOAPdenovo v1.05 software (at a hash length of 73) followed by GapCloser software (at a hash length of 15) (4). The final assembly contains 108 contigs with a total size of 6,126,900 bp and an *N*_50_ contig length of 123 kb; the largest contig assembled measures 318.6 kb. The draft genome (108 contigs) comprising 6,126,900 nucleotides (nt) was annotated with the help of the RAST (Rapid Annotation using Subsystem Technology) system (5) and Aragorn software (6). A total of 5,716 coding sequences (CDS), 2 rRNAs, and 47 tRNAs were predicted.

RAST annotation indicates that *Rhodococcus jostii* strain RHA1 (score, 516), *Rhodococcus erythropolis* strain PR4 (score, 476), and *Rhodococcus opacus* strain B4 (score, 462) are the closest neighbors of the strain BKS 20-38. Genome annotation available at RAST indicates that strain BKS 20-38 contains genes for glycolysis and gluconeogenesis, the tricarboxylic acid (TCA) cycle, and the pentose phosphate pathway. In the RAST annotation, we also found genes for fatty acid metabolic clusters, 3-ketoacyl coenzyme A (3-ketoacyl-CoA) thiolase (EC 2.3.1.16), 3-oxoacyl-[acyl carrier protein] reductase (EC 1.1.1.100), and acyl dehydratase. Also present in the annotation were genes for branched-chain amino acid biosynthesis, 3-isopropylmalate dehydratase small and large subunits (EC 4.2.1.33), 3-isopropylmalate dehydrogenase (EC 1.1.1.85), acetolactate synthase small and large subunits (EC 2.2.1.6), branched-chain amino acid aminotransferase (EC 2.6.1.42), dihydroxy-acid dehydratase (EC 4.2.1.9), ketol-acid reductoisomerase (EC 1.1.1.86), leucine dehydrogenase (EC 1.4.1.9), and threonine dehydratase (EC 4.3.1.19). Genes involved in isoleucine degradation, i.e., those for 3-hydroxyacyl-CoA dehydrogenase (EC 1.1.1.35), 3-ketoacyl-CoA thiolase (EC 2.3.1.16), branched-chain acyl-CoA dehydrogenase (EC 1.3.99.12), branched-chain alpha-keto acid dehydrogenase, E1 component, alpha and beta subunits (EC 1.2.4.4), branched-chain amino acid aminotransferase (EC 2.6.1.42), butyryl-CoA dehydrogenase (EC 1.3.99.2), and enoyl-CoA hydratase (EC 4.2.1.17), were also found in the annotation. Valine degradation genes, i.e., those encoding 3-hydroxyisobutyrate dehydrogenase (EC 1.1.1.31), 3-hydroxyisobutyryl-CoA hydrolase (EC 3.1.2.4), methylmalonate-semialdehyde dehydrogenase (EC 1.2.1.27), acyl-CoA dehydrogenase, short-chain specific (EC 1.3.99.2), and butyryl-CoA dehydrogenase (EC 1.3.99.2), are also present in the genome of strain BKS 20-38.

### Nucleotide sequence accession numbers. 

This whole-genome shotgun project has been deposited at DDBJ/EMBL/GenBank under the accession number AOEX00000000. The version described in this paper is the first version, number AOEX01000000. Genome assembly and annotation data can be downloaded from our genomics web portal at http://crdd.osdd.net/raghava/genomesrs/.
